# In the nose or on the tongue? Contrasting motivational effects of oral and intranasal oxytocin on arousal and reward during social processing

**DOI:** 10.1038/s41398-021-01241-w

**Published:** 2021-02-04

**Authors:** Juan Kou, Chunmei Lan, Yingying Zhang, Qianqian Wang, Feng Zhou, Zhongbo Zhao, Christian Montag, Shuxia Yao, Benjamin Becker, Keith M. Kendrick

**Affiliations:** 1 The Clinical Hospital of Chengdu Brain Science Institute, MOE Key Laboratory for Neuroinformation, University of Electronic Science and Technology of China, Chengdu, Sichuan 611054 China; 2 Department of Molecular Psychology, Institute of Psychology and Education, Ulm University, Ulm, Germany

**Keywords:** Human behaviour, Neuroscience

## Abstract

Intranasal oxytocin exerts wide-ranging effects on socioemotional behavior and is proposed as a potential therapeutic intervention in psychiatric disorders. However, following intranasal administration, oxytocin could penetrate directly into the brain or influence its activity via increased peripheral concentrations crossing the blood–brain barrier or influencing vagal projections. In the current randomized, placebo-controlled, pharmaco-imaging clinical trial we investigated effects of 24IU oral (lingual) oxytocin spray, restricting it to peripherally mediated blood-borne and vagal effects, on responses to face emotions in 80 male subjects and compared them with 138 subjects treated intranasally with 24IU. Oral, but not intranasal oxytocin administration increased both arousal ratings for faces and associated brain reward responses, the latter being partially mediated by blood concentration changes. Furthermore, while oral oxytocin increased amygdala and arousal responses to face emotions, after intranasal administration they were decreased. Thus, oxytocin can produce markedly contrasting motivational effects in relation to socioemotional cues when it influences brain function via different routes. These findings have important implications for future therapeutic use since administering oxytocin orally may be both easier and have potentially stronger beneficial effects by enhancing responses to emotional cues and increasing their associated reward.

## Introduction

The hypothalamic neuropeptide oxytocin (OT) is an important modulator of socioemotional regulation in humans and proposed as a potential therapeutic target for reducing social dysfunction in disorders such as anxiety, autism, and schizophrenia^1–3^. The majority of studies investigating functional effects of OT in both healthy and clinical populations have administered it intranasally. While there have been concerns about whether OT can enter the brain directly via this route^4^, recent animal model^5–10^ and some supporting human studies^11–13^ have produced evidence that it can (see also Quintana et al.^14^), possibly via the olfactory and trigeminal nerves^6^. Since several fMRI studies have compared brain activity changes after both intracerebroventricular and intraperitoneal or intravenous OT administration intranasal OT administration also increases concentrations in peripheral blood, observed functional effects may be contributed to via a peripheral route by the peptide entering the blood and subsequently crossing the blood–brain barrier (BBB) after binding to the immunoglobulin RAGE (receptor for advanced glycation end products)^15,16^. Furthermore, OT can act on its widespread receptors in peripheral organs, such as the heart and gastrointestinal system, and influence brain function via the autonomic nervous system, particularly via vagal or trigeminal stimulation^17^. Thus, while increasing evidence shows that intranasal OT can influence brain function either via transport involving the olfactory and trigeminal nerves or via the BBB after entering the peripheral vasculature or via peripherally mediated autonomic effects, it is still unclear to what extent these different routes mediate its observed effects on brain and behavioral functions.

Research on animal models can administer OT directly into the brain or peripherally via intravenous, intraperitoneal, or oral routes. While central OT administration has produced the most consistent behavioral effects on social behavior in rodents, it can also produce similar functional effects when administered in high doses peripherally^18,19^, despite only a small amount crossing the BBB (around 0.1–0.5%)^20,21^, although behavioral effects differ to some extent^19^. Several fMRI studies have compared brain activity changes after intracerebroventricular, intraperitoneal, or intravenous OT administration. One study in rats reported that changes following intraperitoneal administration, were less extensive than after central administration and restricted to the olfactory bulb and cerebellum^22^, while another found a comparable extent of changes for both routes but with some regional- and sex-specific differences^23^. Finally, a third study in mice found neural activity changes after intranasal administration of OT or vasopressin but none after intravenous administration^24^. Furthermore, experiments using RAGE-knock-out mice support the conclusion that RAGE is important for peripherally-, but not centrally-administered OT to produce effects on social behavior^16^.

In humans, while several studies have reported behavioral^25,26^ or intrinsic neural^12^ effects of intravenously administered OT, one investigating effects of OT on amygdala responses to emotional faces did not, although only a small number of subjects were used and differences between intranasal and intravenous route administration were relatively small and not significant^27^. A study on 6 monkeys using labeled OT reported that following intranasal but not intravenous administration measurable concentrations were found in the tissue of key brain regions such as the orbitofrontal cortex and striatum^7^. However, some caution is needed in interpreting these findings since there was some indication of detectable labeled peptide after intravenous infusion in one animal and it is also possible that the labeling of the peptide might have influenced it being bound by RAGE thereby preventing it from crossing the BBB. On the other hand, a recent study in humans has reported changes in regional cerebral blood flow (rCBF) in many brain regions containing OT receptors following both intranasal and intravenous applications^12^. Furthermore, the latter study reported that increased blood concentrations of OT were correlated with altered rCBF in the amygdala. Thus, the respective importance of the impact of different routes of OT administration on its brain and behavioral effects is still unclear.

Relatively little attention has been paid to the use of an oral route of OT administration as a means of producing both peripherally mediated blood-born and vagal effects on the brain. While intranasally administered OT is targeted to the back of the nose, a certain amount can leak down into the mouth (“trickle-down”). This may explain why several studies have reported extremely high saliva OT concentrations both immediately and lasting for up to 7 h^28–30^ and with no relationship between saliva and blood concentrations^30^. Although it is generally thought that orally administered OT is rapidly degraded in the stomach by endopeptidases, breast milk for example contains significant OT concentrations^31,32^ and studies have shown that RAGE transports OT across the intestinal epithelium into the blood^15^. Furthermore, orally administered OT can increase blood concentrations of the peptide in adult mice^33^ and also alter brain activation and behavior in young mice^34^. In humans, only one study has reported that orally administered OT can increase blood concentrations^34^, either by absorption by blood vessels in the mouth or following ingestion via the gastrointestinal system. Thus, it is possible that oral administration of OT might also produce neural and behavioral effects in humans and, if so, this might open up the possibility of using this route of administration therapeutically.

In the current pre-registered randomized clinical trial, we have therefore used a between-subject design in male subjects to investigate the effects of OT versus placebo (PLC) spray administered orally (lingual spray) rather than intranasally on brain and behavioral responses to emotional faces. A particular focus was on the right amygdala which we have shown previously exhibits reduced responses to all face emotions following intranasal OT^35^. Emotional face tasks are the most frequently used paradigms to demonstrate robust functional effects of intranasal OT^27,35–38^ and where a 24IU dose together with a 45 min time course has been shown to be optimal^39^. We used the same dose (24IU) and bottles for OT/PLC spray as in our previous intranasal experiments and additionally compared the oral with intranasal findings obtained using precisely the same paradigm^35^. Since applying OT via an oral route precludes it entering the brain directly via an olfactory nerve route we hypothesized that this would help identify effects mediated only via peripheral changes in the blood and autonomic nervous system. By comparing the intranasal and oral administration findings we therefore aimed to determine whether peripherally mediated effects following oral administration were similar to those observed following intranasal administration, particularly in terms of effects on right amygdala activation in response to emotional faces and associated arousal.

## Materials and methods

### Participants

Eighty male healthy Chinese University students were recruited for the present study. Five subjects were excluded due to failure to complete the study or excessive head movement (motion >3.0 mm translation or 3° and mean frame-wise displacement (FD < 0.5 mm)^40^ (see CONSORT flowchart Figure S1 for details). Subjects were randomly assigned to receive either OT (*n* = 38, M ± SD, 21.71 ± 1.87 years) or PLC treatment orally (*n* = 37, M ± SD, 22.00 ± 1.93 years) and underwent the experimental paradigm 45 min after administration in line with previous intranasal OT studies^35,39^. We used the a priori option of G*power (version 3.1.9.4) to estimate power using this number of subjects for main analyses of the oral OT trial (two-way ANOVA: treatment × face emotion) with an expected medium effect size (partial eta squared = 0.06) and alpha set to 0.05, which revealed 90% power for observed interactions and 80% for the main effect of treatment. For comparisons between the intranasal and oral trials, a compromise option was used to estimate power of 93% for a two-way ANOVA: route × treatment) with an expected medium effect size (partial eta squared = 0.06). In a pilot study on an independent sample of 25 male subjects, the respective pharmacodynamics of 24IU oral (*n* = 10) and intranasal (*n* = 15) OT were investigated. Following oral OT, there was slower and decreased absorption into the blood compared to the same dose of intranasal OT. Thus, intranasal administration appears to result in slightly faster and also increased OT absorption over time into the blood (for details see SI, Figure S2, and Table S1).

Subject inclusion criteria were the same as in our previous study^35^ (more details see SI). The study had full approval from the local ethics committee of the University of Electronic Science and Technology of China, in accordance with the latest revision of the Declaration of Helsinki and pre-registered as a clinical trial (clinicaltrials.gov NCT04320706). All subjects provided written informed consent and received monetary compensation for participation.

### Experimental procedures and stimuli

The study employed a double-blind, randomized, placebo-controlled, between-subject design. The study was conducted on mornings only to control for any potential circadian changes in OT, with every subject tested between 09:30 and 11:30. Before the experiment, participants completed a number of validated questionnaires to control for pretreatment group differences in potential confounders, including trait anxiety, depression, autism and empathy, and childhood experience (see Supplementary Information). No significant group differences were observed on these scales (see Table S2). After subjects completed the questionnaires, blood samples (10 ml) were collected and they then self-administered the oral treatment (either OT, 24IU; or PLC) by spraying the liquid on the tongue. To match the intranasal protocol, subjects administered the OT/PLC as 6 individual 0.1 ml puffs, one every 30 s. They were instructed not to swallow during the 30 s after each puff in order to give the OT an opportunity to be absorbed by lingual blood vessels. Immediately prior to each new puff of lingual spray subjects were instructed to swallow and thus the OT would also have entered the gastrointestinal system. The OT and PLC spray bottles used were identical to those for intranasal administration and supplied by Sichuan Meike Pharmaceutical Co. Ltd, Sichuan, China. The PLC spray contained identical ingredients (glycerine and sodium chloride) other than OT. In post-treatment interviews, subjects could not identify better than chance whether they had received OT or PLC (*χ*^2^ = 0.89, *p* = 0.48). Thirty minutes after treatment blood samples (10 ml) were collected again to examine changes in OT concentrations following oral treatment and then subjects underwent the implicit face-emotion processing fMRI paradigm (see Figure S3). To control for unspecific mood changes the Positive and Negative Affect Schedule (PANAS)^41^ was administered before and after treatment and following task completion (see Table S3).

A validated implicit face-emotion processing task that has previously demonstrated sensitivity to intranasal OT was employed^35^. Briefly, the event-related paradigm incorporated 104 grayscale facial stimuli displaying happy, neutral, angry, or fearful facial expressions (*n* = 26 per category, 50% female). During the task, subjects were required to judge the gender of the faces to ensure attentive processing. Following the fMRI assessment subjects rated emotional valence, arousal, and intensity of all the stimuli (see Figure S3).

### fMRI data collection and analysis

Blood oxygenation level-dependent (BOLD) contrast time series were acquired using standard sequence parameters on a 3T GE MR750 system. Functional images for task-based data were acquired using EPI sequences (TR = 2000 ms, echo time = 30 ms, flip angle = 90°, FOV = 240 mm × 240 mm, voxel size = 3.75 × 3.75 × 4 mm, resolution = 64 × 64, the number of slices = 39). T1-weighted anatomical images were additionally acquired to improve normalization of the functional images (TR = 6 ms, echo time =2 ms, flip angle = 9°, FOV = 256 mm × 256 mm, voxel size = 1 × 1 × 1 mm, number of slices = 156).

Task-related fMRI data were preprocessed using SPM12^42^, the same standard method as our previous study^35^ (for details, see Supplementary Information). A two-level random-effect general linear model (GLM) analysis was conducted for statistical analyses. First level GLMs for the fMRI data included separate regressors for the four emotional conditions, gender identity rating period, and six movement parameters and appropriate contrasts were subjected to a second-level random-effects analysis. A high-pass filter of 128 s was applied to remove low-frequency drifts. Single-subject contrast images were obtained and subjected to the second-level random-effects analysis.

On the group level the following analyses were conducted: (1) interaction effect between treatment and face emotion via a mixed ANOVA with treatment (PLC, OT) as between-subject factor, face emotions (happy, neutral, angry, and fear) as within-subject factor, and (2) main effect of treatment via direct comparison of the OT and PLC group across all faces using directed two-tailed *t*-test (thresholded at *p* < 0.05). Group-level analyses were conducted using SPM12. Significant clusters were determined using a height threshold of *p* < 0.001 and an extent threshold of *p* < 0.05 with cluster-based family-wise error (FWE) correction on whole-brain level^43,44^.

### Blood sampling and plasma oxytocin measurement

Ten-milliliter venous blood samples were collected into EDTA tubes (2 tubes for pretreatment, 2 tubes for post treatment) and immediately cooled and centrifuged at 1600 × *g* for 15 min at 4 °C. Sampling, handling, and OT assay protocols are given in the supplementary and the assay included a prior extraction step. Note that there has been recent controversy concerning the validity of ELISA results for plasma OT but we incorporated both the recommended extraction step and recovery of spiked samples to address this issue and our basal concentrations are in the normal range^4,45^.

### Statistical analysis of data

Shapiro–Wilks tests implemented on dependent variables of interest indicated that not all were normally distributed for plasma OT concentration, brain and behavioral ratings, therefore nonparametric analyses were performed. We used the R-package for nonparametric ANOVA-type statistics analysis (nparLD function^46^). Where significant interactions occurred post hoc analyses were carried out using two-tailed Mann–Whitney U tests between groups with FDR correction^47^. Measures of effect size for post hoc tests were provided as *r* values^48^. Small, medium, and large effects are 0.1, 0.3, and 0.5, respectively^49^.

### Behavioral rating score data analysis

Effects on stimulus-induced behavioral responses to emotional faces were examined by means of mixed ANOVAs with treatment (PLC, OT) as between-subject factor, face emotion (happy/neutral/angry/fear) as within-subject factor using the R-package^47^ for nonparametric ANOVAs. FDR-corrected post hoc comparisons were used to explore significant (*p* < 0.05) interactions.

### Changes in plasma oxytocin concentrations and associations with neural and behavioral responses

Two-tailed Mann–Whitney U tests between groups were conducted to analyze whether mean concentrations of plasma OT were increased following OT vs PLC using percentage changes from baseline (i.e., relative change). Correlation analyses (Spearman) were used to investigate the relationship between altered OT concentrations and significant effects of OT on behavioral ratings and neural activation changes. Beta values of brain activation were extracted by marsbar (Marsbar, http://marsbar.sourceforge.net) for examining associations with percentage OT blood level changes. Furthermore, in line with our hypotheses, a mediation analysis was conducted to investigate whether (1) the percentage OT concentration change mediated the effect of treatment on neural responses and (2) functional brain changes mediated the effects of treatment on the behavioral level by means of a bootstrapping method^50^.

### Comparison between neural and behavioral responses following oral or intranasal OT

To investigate administration-route dependent effects of OT we additionally compared data from the current study (oral administration) with that from our previous 24IU OT intranasal administration one using the same implicit face processing paradigm (day 1 acute treatment—see Kou et al.^35^). The pooled data were analyzed using mixed ANOVAs with the between-subject factors route (intranasal/oral) and treatment (OT and PLC) and within factor face emotion (neutral/fear/angry/happy). Based on our a priori regional hypothesis on the right amygdala and the observed modulatory effect of OT on the right putamen, beta values were extracted from atlas-defined masks (masks defined by the Brainnetome Atlas^51^) and served as dependent variables. FDR-corrected post hoc comparisons were used to explore significant (*p* < 0.05) interactions.

Differences in stimulus-induced behavioral responses (valence, intensity, and arousal ratings) to emotional faces were examined by means of mixed ANOVAs with treatment (OT/PLC) and route (Intranasal/Oral) as between-subject and face emotion (happy, neutral, angry, and fear) as within-subject factors. FDR-corrected post hoc comparisons were used to explore significant (*p* < 0.05) interactions.

## Results

### Effects of oral OT on neural and behavioral responses to face emotions

**Fig. 1:**
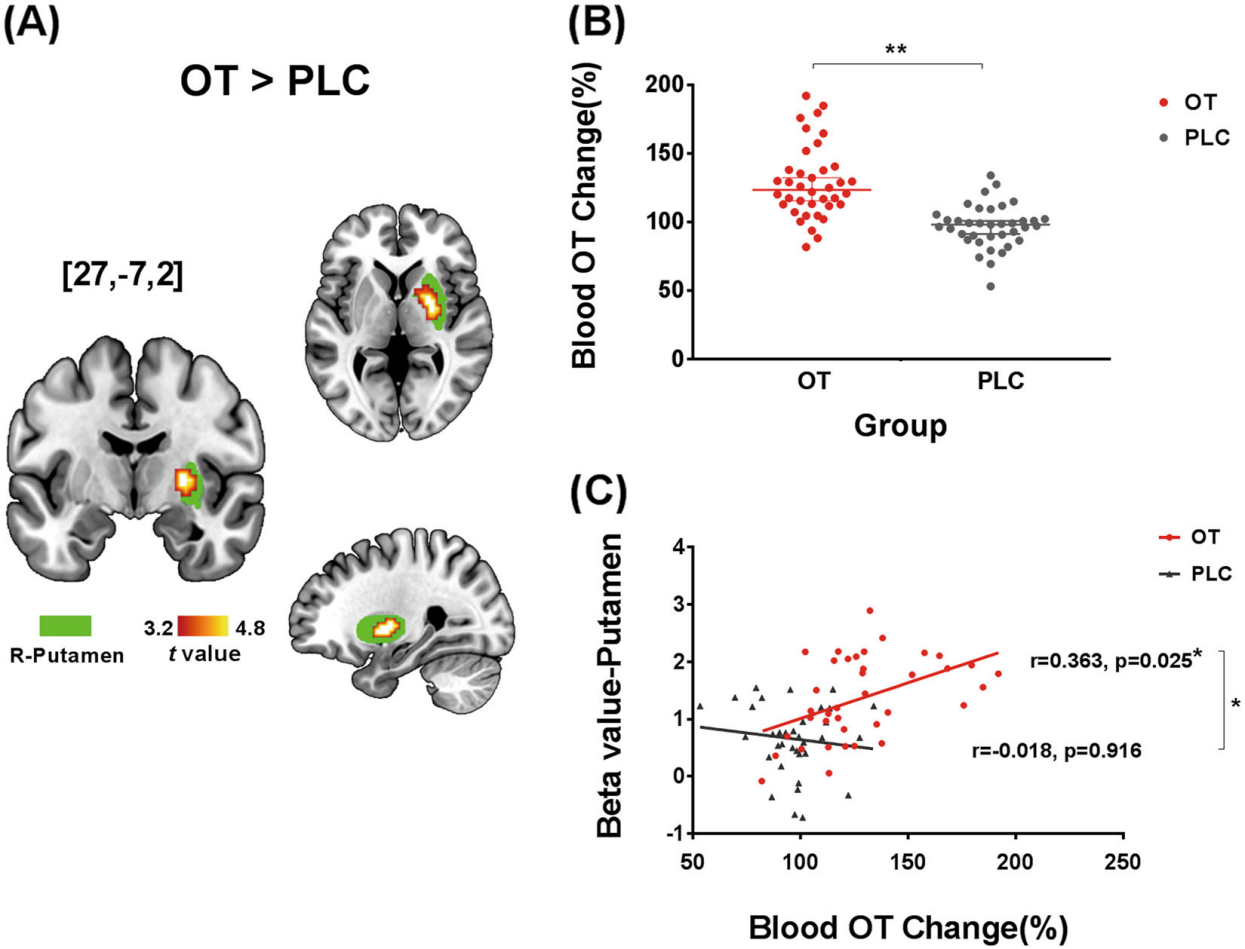
Main effect of oral oxytocin (OT) on putamen responses and the relationship between OT blood level change and brain activity. **A** The whole-brain voxel-wise analysis revealed a significant main effect of treatment in the right putamen reflecting that OT increased activation in this region relative to PLC (the cluster is presented in color, displayed at *p* < 0.05_FWE_); the overlaying mask in green represents the right putamen from the Brainnetome Atlas^51^ which was subsequently used as atlas-based independent region of interest (ROI) to extract beta-estimates for further analyses. **B** Group difference of OT concentration change. **C** Correlation between putamen activation to all faces and percentage concentration change of plasma oxytocin.

While no significant treatment × face-emotion interaction effect was observed, the results revealed a significant main effect of treatment (PLC versus OT) in the right putamen (peak MNI, *xyz* = [27, −7, 2], *t*_1.73_ = 4.76, *p*_FWE cluster_ = 0.021, *k* = 55), indicating that oral OT significantly increased putamen responses to facial stimuli regardless of emotion (Fig. 1A).

**Fig. 2:**
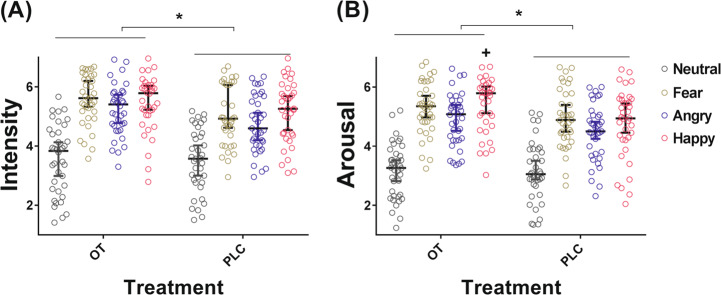
Effects of oral oxytocin (OT) on intensity and arousal ratings for different face emotions. **A** OT increased intensity ratings to all face emotions. **B** OT increased emotional arousal ratings to all face emotions but to angry and happy faces more than others. PLC placebo. *p_FDR_ < 0.05 (all faces), +*p*_FDR_ < 0.05 (for specific emotional faces). Bars indicate 95% confidence intervals and medians.

For emotional valence, intensity and arousal ratings there were only significant main effects of treatment for intensity and arousal (intensity: F_(1.73)_ = 5.21, *p* = 0.022; arousal: F_(1.73)_ = 5.19, *p* = 0.023; valence *p* = 0.224) indicating that OT increased intensity and arousal ratings for all face stimuli. There were also significant treatment × face-emotion interactions for arousal ratings (arousal: F_(2.219)_ = 3.47, *p* = 0.025; valence *p* = 0.081; intensity *p* = 0.176). Post hoc comparisons of interaction effect for arousal ratings revealed the OT group gave increased arousal ratings to happy and angry faces (happy: *p*_FDR_ = 0.016, *r* = 0.329; angry: *p* = 0.05, *p*_FDR_ = 0.1, *r* = 0.226 does not pass correction) relative to the PLC group (see Fig. 2).

### Effects of oral OT on plasma concentrations and correlations with brain and behavioral responses

There was no between-group difference in baseline OT concentrations in samples taken prior to treatment (*p* = 0.522). Following oral administration, OT concentrations increased significantly by 30 min compared with baseline (median increase = 2.30 pg/ml, *p* < 0.001 and median % change = +25.32%, *p* < 0.001, see Fig. 1B—similar to our pilot study see Figure S2). Based on the main effect of treatment the beta values from an independently (atlas) defined right putamen mask were extracted. There was a significant positive correlation between percentage plasma OT changes and putamen responses to face-emotion stimuli in the oral OT group (*r* = 0.363, *p* = 0.025) (Fig. 1C). In addition, beta values were similarly extracted from an independently defined right amygdala mask but no correlation with percent change in plasma OT concentrations was found (*r* = 0.271, *p* = 0.10). No significant correlations between plasma OT changes and behavioral ratings were found (*p*s > 0.364).

### Mediation analysis of relationships between OT changes and neural and behavioral effects

**Fig. 3:**
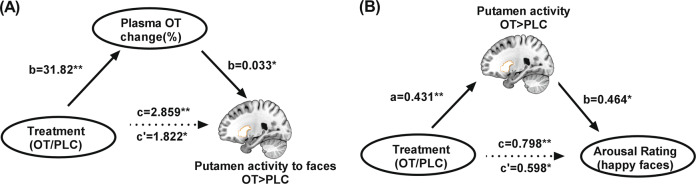
Mediation analysis for putamen responses to faces and oxytocin (OT) concentrations and behavior ratings. **A** Plasma OT concentration % change partially mediated the oxytocin treatment effect on putamen responses to all face emotions. **B** Putamen responses partially mediated the OT treatment effect on arousal rating to happy faces. PLC placebo. *p < 0.05, **p < 0.01.

Mediation analysis revealed that oral OT treatment increased plasma OT percentage change (path *a* = 31.82, *p* < 0.001) and putamen responses to face emotions (path *c* = 2.859, *p* < 0.001). Plasma OT percentage change was positively associated with putamen responses to emotional faces independent of treatment (path *b* = 0.033, *p* = 0.020) and when included in the model as a mediator the direct effect of OT treatment on putamen responses to face emotions was attenuated (path *c*′ = 1.822, *p* = 0.016). These results indicate a partial mediation of plasma OT percentage change on the relationship between oral OT treatment and putamen responses to emotional faces (indirect effect = 1.04, 95% CI = [0.08, 0.58], bootstrap = 5000, see Fig. 3A). A further mediation analysis of putamen activation and its relationship with OT treatment and behavioral responses (arousal/intensity ratings) to all emotional faces was not significant, however, a partial mediation effect was found when we restricted it to happy faces (indirect effect = 0.20, 95% CI = [0.04, 0.48], bootstrap = 5000). In the model, OT treatment increased putamen responses (path *a* = 0.431, *p* = 0.003) and arousal ratings (path *c* = 0.798, *p* = 0.003) to happy faces and putamen activity was significantly independently associated with changes in arousal ratings (path *b* = 0.464, *p* = 0.036). When putamen activity was added to the model, the correlation between OT treatment and arousal ratings was attenuated (path *c*′ = 0.598, *p* = 0.031) (see Fig. 3B). Results therefore indicate that by influencing the OT concentration in blood, OT treatment partially mediated the putamen response to all emotional faces and that effects of OT treatment specifically on the putamen response to happy faces partially mediated increased arousal ratings for happy faces.

### Region of interest comparison between intranasal and oral OT effects on neural responses to face emotions

**Fig. 4:**
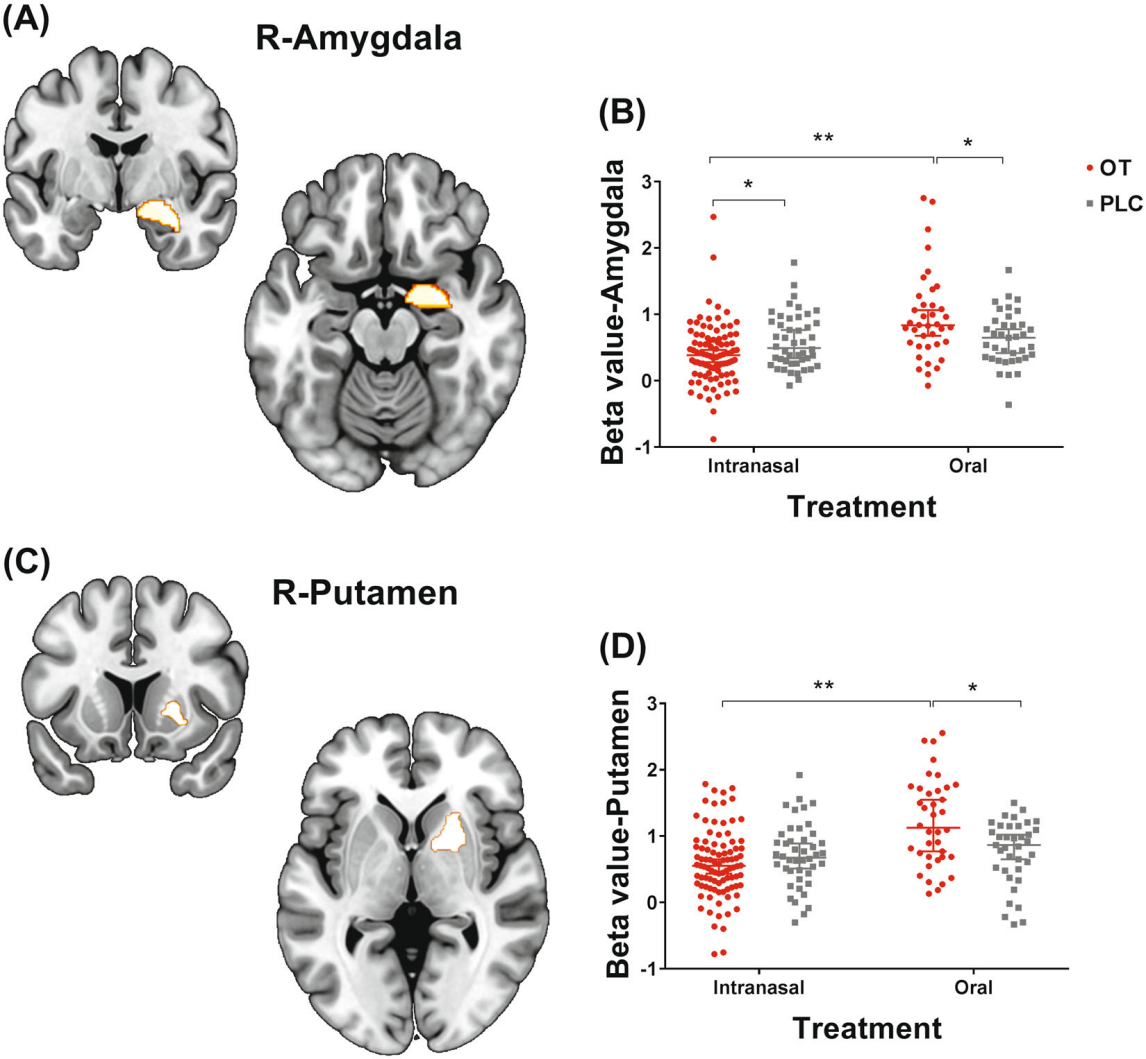
Region of interest (ROI) analysis comparing effects of oral and intranasal oxytocin (OT) on amygdala and putamen responses. **A** right amygdala mask from the Brainnetome Atlas. **B** Oral OT significantly increased activation of the right amygdala, while intranasal oxytocin decreased it. **C** Right putamen mask from Brainnetome Atlas **D** oral oxytocin increased the activation of right putamen to all face emotions but intranasal OT had no effect. PLC placebo. *p_FDR_ < 0.05, **p_FDR_ < 0.01.

Based on our a priori hypothesis amygdala and putamen responses to face emotions following oral and intranasal OT administration were analyzed using a mixed ANOVA with treatment (OT/PLC) and administration route (intranasal/oral) as between and face emotion (neutral/happy/fear/angry) as within factors. There was a significant treatment × route interaction (amygdala: F_(1.209)_ = 10.86, *p* = 0.001; putamen: F_(1.209)_ = 7.993, *p* = 0.005), but no treatment × route × face-emotion interaction (amygdala: *p* = 0.640; putamen: *p* = 0.170). Post hoc comparisons revealed that the intranasal OT group exhibited significantly decreased activation of right amygdala compared with the intranasal PLC group to all faces (*p*_FDR_ = 0.033, *r* = 0.207) but not for right putamen (*p* = 0.268), while the oral OT group exhibited significantly increased activation of right amygdala (*p*_FDR_ = 0.033, *r* = 0.268) and right putamen (*p*_FDR_ = 0.018, *r* = 0.302) compared with oral PLC group. This finding was further validated by a significant difference between the two OT treatment groups for both right amygdala (intranasal vs oral OT, *p*_FDR_ < 0.001, *r* = 0.434) and putamen (*p*_FDR_ < 0.001, *r* = 0.411), while there was no significant difference between the two PLC groups (*p*s > 0.223) (see Fig. 4).

### Comparison between intranasal and oral OT on behavioral ratings of emotional faces

**Fig. 5:**
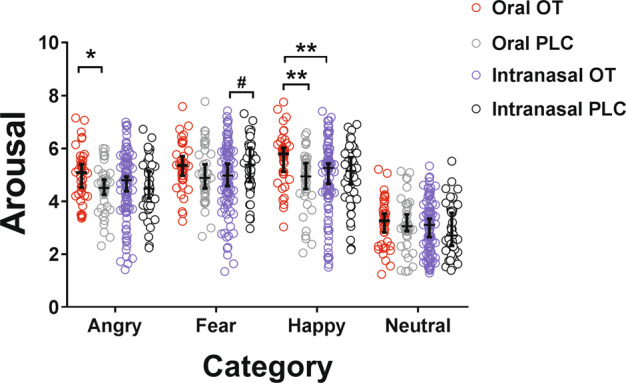
Comparisons of arousal ratings for face emotions between oral and intranasal oxytocin (OT) administration. ^#^*p* < 0.1, *p < 0.05, **p < 0.01. Bars indicated 95% confidence intervals and medians.

For emotional valence, intensity and arousal ratings, mixed ANOVA analysis revealed no significant main effects of treatment or route (*p*s > 0.063) but a significant main effect of face emotion for valence, intensity and arousal ratings (valence: F_(2.232)_ = 868.65, *p* < 0.001; intensity: F_(2.232)_ = 226.27, *p* < 0.001, *η*^2^_*p*_ = 0.550; arousal: F_(2.232)_ = 275.46, *p* < 0.001). There was a significant treatment × route × face-emotion interaction for arousal ratings (F_(2.232)_ = 3.44, *p* = 0.023) but not for valence or intensity (valence: *p* = 0.073; intensity: *p* = 0.077). Post hoc comparisons of treatment × route × face for arousal ratings revealed that the oral OT group exhibited increased ratings for happy and angry faces relative to the oral PLC one (happy: *p* = 0.004, *p*_FDR_ = 0.016, *r* = 0.439; angry: *p* = 0.050, *p*_FDR_ = 0.20, *r* = 0.225) and increased ratings for happy faces relative to the intranasal OT group (happy: *p*_FDR_ = 0.019, *r* = 0.228). The intranasal OT group exhibited a marginal decrease in ratings for fear faces (*p* = 0.08) but there were no significant group differences for other face emotions (*p*s > 0.10) (see Fig. 5).

## Discussion

Overall, our findings demonstrate that administering OT to male subjects orally as a lingual spray increased responsivity of the putamen—a key region in the brain reward system—and corresponding intensity and arousal ratings for all emotional faces. Furthermore, a mediation analysis revealed that the increased blood OT concentrations partially mediated enhanced putamen responses to all face emotions and that the effects of OT on putamen responses to happy faces partially mediated increased arousal ratings. A direct comparison of amygdala activation between subjects given OT by oral and intranasal routes revealed that whereas oral administration increased amygdala responses to all face emotions it decreased them following intranasal administration and these differences in treatment route were significant. Similarly, while oral OT increased putamen responses to all face emotions, intranasal OT had no effect. In line with the neural findings, behavioral responses to emotional faces were significantly increased by oral OT relative to intranasal treatment.

Overall the profile of neural and behavioral responses to face emotions produced following 24IU oral administration of OT to males suggests that it enhances both rewarding and arousal effects of all faces, although particularly happy ones. By contrast, the effects of the same OT dose administered via an intranasal route are suggestive of more of an anxiolytic effect with amygdala responses to all faces being reduced although with corresponding reductions in arousal mainly for fear faces. Both of these contrasting effects could be of potential therapeutic benefit dependent upon whether the objective is to enhance positive motivation towards social stimuli or to reduce anxiety in response to threatening ones.

By administering OT orally using the same spray device and dose and treatment timing as for intranasal treatment we aimed to provide a reasonable comparison of the functional effects produced by the two different routes of administration. As we had anticipated, the profiles of altered blood OT concentrations following the oral and intranasal treatments were broadly similar, although concentrations were higher following the intranasal dose, and both routes would have resulted in OT entering the gastrointestinal system, although the assumption would be that this would have been higher for the oral dose. Nevertheless, the neural and behavioral responses to OT administered by the two different routes were very different. While it is possible that this reflects the fact that OT administered intranasally, unlike orally, may additionally produce effects by penetrating into the brain via the olfactory and trigeminal nerves^6,14^, we cannot entirely rule out that there might also have been some differential peripheral blood-borne or autonomic effects produced by the two administration routes. The relative amounts of peptide being absorbed into the blood and gastrointestinal system in the two routes of administration could, for example, have contributed to these differences.

Peripherally mediated effects of exogenously administered OT by either oral, intravenous or intranasal routes could be produced by a number of different mechanisms. The main focus of previous studies addressing this issue has been on increased blood concentrations of OT resulting in it modulating brain function after crossing the BBB, and we now know that there is a molecular mechanism for this transport involving RAGE^15,16^. However, OT could also be influencing brain function without crossing the BBB via its peripheral receptors to promote vagal- or trigeminal-mediated effects. Our current findings showing that increased OT concentrations in blood partially mediate enhanced putamen responses to face emotions indicate that effects may at least be partly mediated by the peptide crossing the BBB. However, the mediation analysis suggested that >60% of the mediation was via another route and therefore OT effects via the vagus or trigeminal or other routes might be influential as well. It would be of interest to compare the effects of oral and intravenous administration of OT in this respect since the latter may have less influence on the gastric vagal system, for example. We also found no significant relationship at all between OT changes and increased amygdala responses to faces. This latter observation is contrary to a recent study on 17 male subjects reporting an association between increased peripheral OT concentrations and reduced regional cerebral blood flow although this was at a shorter time course following OT administration and did not involve a task^12^.

At this point, we can only speculate on the mechanisms whereby exogenously administered OT can produce different effects on brain and behavior via oral and intranasal routes. One possibility is that different amounts of OT bound to RAGE in order the cross the blood–brain barrier^15^ can influence which OT receptors it can bind to or perhaps which type of Gα protein is formed. The latter can determine whether OT increases or decreases cell activity^52^. Another possibility is that when OT additionally enters the brain via the olfactory or trigeminal nerves following intranasal administration it has a different effect from that entering the brain via the blood after binding to RAGE. For example, opto-genetic approaches in mice have demonstrated that OT released within the brain produces predominantly inhibitory effects in cortical circuitry in males via specific receptors on GABA interneurons, although interestingly not in females^53^. There also remains the possibility of different inverted U-shaped dose-dependent profiles of OT effects on brain and behavioral responses, particularly involving the social reward system (see Borland et al.^54^).

The current study used only male subjects and there is increasing evidence for sex-differences in both neural and behavioral responses^55^. Most notable in the context of the current findings is that while a 24IU dose of intranasal OT decreases amygdala responses to fear or all face emotions and arousal in males^27,35^, in females it enhances amygdala responses to fear faces^35^. Furthermore, we also found that in men oral OT produced significantly enhanced putamen responses and arousal ratings compared to intranasal OT for all face emotions, whereas a previous study found the same enhanced responses to happy faces after 24IU intranasal OT only in females^56^. Indeed, we found that oral OT-treatment effects on increasing putamen responses to happy faces in men partially mediated increased arousal ratings emphasizing the greater association between OT effects on the putamen and enhanced arousal to positive valence stimuli. Thus, administering OT orally in males may produce a similar profile of neural and behavioral responses as intranasal OT does in females, however, this needs to be explored in further experiments which also take into account possible sex-dependent inverted U-shaped response profiles (see Borland et al.^54^).

The lingual spray protocol we used resulted in OT being absorbed both by lingual blood vessels and subsequently in the gastrointestinal system after being swallowed. At this point it is not clear whether oral dosing in the form of a capsule, which would prevent absorption via blood vessels in the mouth, might have similar effects to the lingual spray. Administering OT orally in capsule form can both increase blood OT concentrations and promote neural effects in mice, presumable via RAGE-mediated intestinal transport^15,16^. Nevertheless, to the best of our knowledge this is the first study of functional effects of OT given orally as a lingual spray and to demonstrate that it produces a similar general profile of increased blood concentrations as the same 24IU dose of intranasal spray in terms of duration (around 1 h), although the peak absorption is slightly slowed and the magnitude of changes is significantly smaller. This opens up the possibility of using OT therapeutically as a lingual spray which may be more easily administered than by intranasal or intravenous routes.

Several limitations should be acknowledged in the current study. First, only male subjects were included in line with most other recent studies investigating kinetics and routes of administration effects of OT^11,12,27,39^. However, there is increasing evidence for differential neural and behavioral responses to OT during processing of social stimuli^55–57^ and it will be important to establish to what extent these are contributed to by possible sex-differences in the relative effects of different routes of administration of the peptide. Second, while the use of OT administration via lingual spray should have produced a broadly similar pattern of peripherally mediated effects as that of the intranasal one, they are clearly not identical and notably the latency and magnitude of increased blood OT concentrations were significantly different with vagal effects following gastrointestinal absorption possibly being higher. Thus, different lingual spray doses might have resulted in different effects and it will be important for further studies to investigate potential dose–response effects. On the other hand, it seems unlikely that achieving similar absorption rates to an oral dose into the blood using a lower intranasal dose might have produced a similar profile of responses since a previous study found no effects at all of a lower 12IU dose on amygdala responses to fear faces despite much reduced increases in plasma OT concentrations compared with a 24IU dose^39^. Future research using selective brain-penetrant and non-brain-penetrant antagonists to dissect the potential contributions of peripherally mediated effects of OT, such as gastric vagal effects, will hopefully help to further elucidate the mechanisms involved. Thirdly, the current study did not consider the possibility of different time courses of potential effects of oral and intranasal OT, although a previous study found that intranasal effects of 24IU OT on reduced amygdala responses to fear faces occur maximally from 45 to 75 min after administration but not earlier. No evidence for any time-dependent differences in the pattern of responses was found^39^. Finally, the current study did not include any measures which would have permitted an assessment of potential vagal- or trigeminal-mediated effects of OT following oral administration.

In summary, the current study has demonstrated that administering OT via an oral (lingual) route produces increased responses in the brain reward system to face emotions and associated arousal and that neural responses are partially mediated via increased OT concentrations in blood. This contrasts markedly with identical administration via an intranasal route where there is no such effect on the reward system in males and where the amygdala response to face emotions and corresponding arousal ratings are decreased whereas following oral OT both are increased. These findings suggest that intranasal and oral routes of exogenous OT administration may produce different mechanisms of action resulting in contrasting motivational responses to socioemotional cues. In addition, our findings open up the potential for oral OT as a potential therapeutic route which may have advantages in terms of ease of administration, particularly in children.

## Supplementary information

Supplemental information

## References

[1] Kendrick, K. M., Guastella, A. J., & Becker, B. in *Behavioral Pharmacology of Neuropeptides: Oxytocin* 321–348 (Springer, 2017).

[2] Young LJ, Barrett CE (2015). Can oxytocin treat autism?. Science.

[3] Meyer-Lindenberg A, Domes G, Kirsch P, Heinrichs M (2011). Oxytocin and vasopressin in the human brain: social neuropeptides for translational medicine. Nat. Rev. Neurosci..

[4] Leng G, Ludwig M (2016). Intranasal oxytocin: myths and delusions. Biol. Psychiatry.

[5] Beard R, Singh N, Grundschober CD, Gee AW, Tate E (2018). High-yielding 18 F radiosynthesis of a novel oxytocin receptor tracer, a probe for nose-to-brain oxytocin uptake in vivo. Chem. Commun..

[6] Lee MR (2018). Oxytocin by intranasal and intravenous routes reaches the cerebrospinal fluid in rhesus macaques: determination using a novel oxytocin assay. Mol. Psychiatry.

[7] Lee MR (2020). Labeled oxytocin administered via the intranasal route reaches the brain in rhesus macaques. Nat. Commun..

[8] Modi ME, Connor-Stroud F, Landgraf R, Young LJ, Parr LA (2014). Aerosolized oxytocin increases cerebrospinal fluid oxytocin in rhesus macaques. Psychoneuroendocrinology.

[9] Neumann ID, Maloumby R, Beiderbeck DI, Lukas M, Landgraf R (2013). Increased brain and plasma oxytocin after nasal and peripheral administration in rats and mice. Psychoneuroendocrinology.

[10] Smith AS, Korgan AC, Young WS (2019). Oxytocin delivered nasally or intraperitoneally reaches the brain and plasma of normal and oxytocin knockout mice. Pharmacol. Res..

[11] Paloyelis Y (2016). A spatiotemporal profile of in vivo cerebral blood flow changes following intranasal oxytocin in humans. Biol. Psychiatry.

[12] Martins DA (2020). Effects of route of administration on oxytocin-induced changes in regional cerebral blood flow in humans. Nat. Commun..

[13] Striepens N (2013). Elevated cerebrospinal fluid and blood concentrations of oxytocin following its intranasal administration in humans. Sci. Rep..

[14] Quintana DS (2020). Advances in the field of intranasal oxytocin research: lessons learned and future directions for clinical research. Mol. Psychiatry.

[15] Yamamoto Y (2020). RAGE regulates oxytocin transport into the brain. Commun. Biol..

[16] Yamamoto, Y. et al. Vascular RAGE transports oxytocin into the brain to elicit its maternal bonding behaviour in mice. *Commun. Biol.* **2**, 10.1038/s42003-019-0325-6 (2019). 10.1038/s42003-019-0325-6 PMC6389896 30820471

[17] Carter CS (2014). Oxytocin pathways and the evolution of human behavior. Annu. Rev. Psychol..

[18] Ring RH (2006). Anxiolytic-like activity of oxytocin in male mice: Behavioral and autonomic evidence, therapeutic implications. Psychopharmacology.

[19] Sakamoto, T., Sugimoto, S. & Uekita, T. Effects of intraperitoneal and intracerebroventricular injections of oxytocin on social and emotional behaviors in pubertal male mice. *Physiol. Behav*. 212 (2019). 10.1016/j.physbeh.2019.112701 31629768

[20] Mens WBJ, Witter A, Van Wimersma Greidanus TB (1983). Penetration of neurohypophyseal hormones from plasma into cerebrospinal fluid (CSF): Half-times of disappearance of these neuropeptides from CSF. Brain. Res..

[21] Ermisch A (1985). On the blood-brain barrier to peptides: accumulation of labelled vasopressin, DesGlyNH2-vasopressin and oxytocin by brain regions. Endocrinol. Exp..

[22] Ferris, C. F. et al. Distinct BOLD Activation Profiles Following Central and Peripheral Oxytocin Administration in Awake Rats. *Front. Behav. Neurosci*. **9**, 10.3389/fnbeh.2015.00245 (2015). 10.3389/fnbeh.2015.00245 PMC4585275 26441574

[23] Dumais KM, Kulkarni PP, Ferris CF, Veenema AH (2017). Sex differences in neural activation following different routes of oxytocin administration in awake adult rats. Psychoneuroendocrinology.

[24] Galbusera A (2017). Intranasal oxytocin and vasopressin modulate divergent brainwide functional substrates. Neuropsychopharmacology.

[25] Hollander E (2003). Oxytocin infusion reduces repetitive behaviors in adults with autistic and Asperger’s disorders. Neuropsychopharmacology.

[26] Hollander E (2007). Oxytocin increases retention of social cognition in autism. Biol. Psychiatry.

[27] Quintana DS (2016). Low dose intranasal oxytocin delivered with Breath Powered device dampens amygdala response to emotional stimuli: a peripheral effect-controlled within-subjects randomized dose-response fMRI trial. Psychoneuroendocrinology.

[28] Daughters K (2015). Salivary oxytocin concentrations in males following intranasal administration of oxytocin: a double-blind, cross-over study. PLoS ONE.

[29] van IJzendoorn MH, Bhandari R, van der Veen R, Grewen KM, Bakermans-Kranenburg MJ (2012). Elevated salivary levels of oxytocin persist more than 7 h after intranasal administration. Front. Neurosci..

[30] Quintana DS (2018). Saliva oxytocin measuresDESP do not reflect peripheral plasma concentrations after intranasal oxytocin administration in men. Horm. Behav..

[31] Takeda S, Kuwabara Y, Mizuno M (1986). Concentrations and origin of oxytocin in breast milk. Endocrinol. Jpn..

[32] Petersson M, Hulting AL, Andersson R, Uvnäs-Moberg K (1999). Long-term changes in gastrin, cholecystokinin and insulin in response to oxytocin treatment. Neuroendocrinology.

[33] Maejima Y (2020). Oral oxytocin delivery with proton pump inhibitor pretreatment decreases food intake. Peptides.

[34] Groot ANJAD (1995). Bioavailability and pharmacokinetics of sublingual oxytocin in male volunteers. J. Pharm. Pharmacol..

[35] Kou J (2020). A randomized trial shows dose-frequency and genotype may determine the therapeutic efficacy of intranasal oxytocin. Psychol. Med..

[36] Kirsch P (2005). Oxytocin modulates neural circuitry for social cognition and fear in humans. J. Neurosci..

[37] Koch SBJ (2016). Intranasal oxytocin normalizes amygdala functional connectivity in posttraumatic stress disorder. Neuropsychopharmacology.

[38] Domes G (2007). Oxytocin attenuates amygdala responses to emotional faces regardless of valence. Biol. Psychiatry.

[39] Spengler FB (2017). Kinetics and dose dependency of intranasal oxytocin effects on amygdala reactivity. Biol. Psychiatry.

[40] Power JD, Barnes KA, Snyder AZ, Schlaggar BL, Petersen SE (2012). Spurious but systematic correlations in functional connectivity MRI networks arise from subject motion. NeuroImage.

[41] Watson D, Clark LA, Tellegen A (1988). Development and validation of brief measures of positive and negative affect: the PANAS scales. J. Pers. Soc. Psychol..

[42] Friston KJ (1994). Statistical parametric maps in functional imaging: a general linear approach. Hum. Brain Mapp..

[43] Flandin G, Friston K (2019). Analysis of family-wise error rates in statistical parametric mapping using random field theory. Hum. Brain Mapp..

[44] Woo CW, Krishnan A, Wager TD (2014). Cluster-extent based thresholding in fMRI analyses: Pitfalls and recommendations. NeuroImage.

[45] McCullough ME, Churchland PS, Mendez AJ (2013). Problems with measuring peripheral oxytocin: Can the data on oxytocin and human behavior be trusted?. Neurosci. Biobehav. Rev..

[46] Noguchi, K., Gel, Y. R., Brunner, E. & Konietschke, F. nparLD: an R software package for the nonparametric analysis of longitudinal data in factorial experiments. *J. Stat. Softw*. **50** (2012).

[47] Cramer AOJ (2016). Hidden multiplicity in exploratory multiway ANOVA: prevalence and remedies. Psychon. Bull. Rev..

[48] Cooper, H. & Hedges, L. V. *The Handbook of Research Synthesis* 1560–1562 (Russell Sage Foundation, 1994).

[49] Coolican, H. *Research Methods and Statistics in Psychology* (Routledge, 2018).

[50] Preacher KJ, Hayes AF (2008). Asymptotic and resampling strategies for assessing and comparing indirect effects in multiple mediator models. Behav. Res. Methods.

[51] Fan L (2016). The human brainnetome atlas: a new brain atlas based on connectional architecture. Cereb. Cortex.

[52] Jurek B, Neumann ID (2018). The oxytocin receptor: from intracellular signaling to behavior. Physiol. Rev..

[53] Li K, Nakajima M, Ibañez-Tallon I, Heintz N (2016). A cortical circuit for sexually dimorphic oxytocin-dependent anxiety behaviors. Cell.

[54] Borland JM, Rilling JK, Frantz KJ, Albers HE (2019). Sex-dependent regulation of social reward by oxytocin: an inverted U hypothesis. Neuropsychopharmacology.

[55] Gao S (2016). Oxytocin, the peptide that bonds the sexes also divides them. PNAS.

[56] Lieberz J (2019). Kinetics of oxytocin effects on amygdala and striatal reactivity vary between women and men. Neuropsychopharmacology.

[57] Luo L (2017). Sex-dependent neural effect of oxytocin during subliminal processing of negative emotion faces. NeuroImage.

