# Supplementary material for: In the nose or on the tongue? Contrasting motivational effects of oral and intranasal oxytocin on arousal and reward during social processing
Source: Transl Psychiatry. 2021 Feb 4;11:94. doi: 10.1038/s41398-021-01241-w (PMC7862637; doi:10.1038/s41398-021-01241-w)
Supplement: Supplementary file 1 — Supplemental information [file 41398_2021_1241_MOESM1_ESM.docx]

**Supplementary Information**

**Kou et al., “In the nose or on the tongue? Contrasting motivational effects of oral and intranasal oxytocin on arousal and reward during social processing.”**

**Supplementary Methods and Results**

**Supplementary Tables**

**Supplementary Figures**

**Supplementary Methods and Results**

**Participants**

80 male healthy Chinese University students were recruited for the present study in the oral OT administration experiment. All subjects self-reported being free of a history of, or current, medical or psychiatric disorder and were currently not taking any medication. Subjects were required to abstain from alcohol, caffeine or nicotine in the 24 hours before the experiment. In total, five subjects were excluded due to failure to complete the study or excessive head movement (motion > 3.0mm translation or 3° and mean frame-wise displacement (FD < 0.5 mm) (see CONSORT flowchart presented in Figure. S1). The enrollment criteria for the 138 male participants in intranasal OT study were identical to the one in the present oral experiment (more details see - Kou, et al., 2020)

**Methods and Results for profiling OT concentrations in blood following oral or intranasal OT**

In a pilot study examining the pharmacokinetics of oral versus intranasal OT administration in an independent sample (n = 25 healthy subjects) OT concentrations were measured in blood samples taken every 15 minutes before (baseline: -15 min and 0) and following (+15, 30, 45, 60, 75 and 90 mins) oral or intranasal OT. 10 subjects were randomly assigned for oral and 15 subjects for intranasal administration. In the main study blood samples were taken from the 80 subjects immediately before oral OT or PLC administration and 30 min after it. Each subject’s venous blood was collected into two 5 ml EDTA tubes which were immediately kept on ice and centrifuged at 1600g for 15 min at 4°C within half an hour of collection. Plasma was immediately aliquoted into chilled Eppendorf tubes and stored at -80°C until oxytocin analysis. All samples were analyzed within 3 months of collection.

Oxytocin concentrations were analyzed in 1ml plasma samples and measured in triplicate using a commercial ELISA assay (ENZO Life Sciences, 60 Executive Boulevard, Farmingdale, New York 11735). A standard prior extraction step was performed in accordance with the manufacturers recommended protocol and spiked samples (with 100pg/ml oxytocin added) were included in each assay to calculate extraction efficiency which was 96.6%. The extraction step incorporated a 2-fold concentration of samples using a vacuum concentrator (Concentrator plus, Eppendorf, Germany) resulting in a detection sensitivity of 3pg/ml. The majority of samples had detectable concentrations (> 98%) and those which did not were assigned the minimum detection value. The intra- and inter-assay coefficients of variation were 8.83% and 6.84% respectively. The manufacturer’s reported cross-reactivity of the antibody with related neuropeptides, such as vasopressin and vasotocin is <0.01%.

The area under the curve (AUC) represents how much of an administered drug is in the plasma over a given time. The Trapezoid rule calculation divides the concentration-time plot up into a series of trapezoids and then the sum of all the areas of the trapezoids can be calculated. The AUC from 0 to 1.5 hours (AUCt) was determined using GraphPad Prism version 7.04 for Windows (www.graphpad.com). Two-tailed t-tests were used to compare the AUCt Cmax and Tmax between oral and intranasal routes and the R PK package^1^ to retest results of pharmacokinetic parameters.

The profiles of OT blood concentrations changes after oral and intranasal OT administration are shown in Figure.S2. Pharmacokinetic parameters for OT after intranasal or oral administration are shown in Table S1. All subjects in the oral OT group had a smaller AUCt compared with the intranasal OT group (p=0.01), lower peak plasma concentrations (Cmax) (p=0.004) and were slower to reach peak concentrations (Tmax) (p=0.05) after administration.

**Experimental procedure for the oral OT administration part**

Before the experiment, participants were required to complete a number of validated questionnaires including trait anxiety, depression, autism and empathy and childhood experience: State-Trait Anxiety Inventory (STAI)^1^, Liebowitz Social Anxiety Scale (LSAS)^2^, Beck Depression Inventory (BDI-II)^3^, Autism-Spectrum Quotient (AQ)^4^, Social Responsiveness Scale second version (SRS-2)^5^, Interpersonal Reactivity Index (IRI) ^6^ and Childhood Trauma Questionnaire (CTQ)^7^(see Table S2). After subjects completed the questionnaires, blood samples (10 ml) were collected before the treatment administration. Subjects next administered the oral treatment (either OT, 24 IU; or PLC) lingually, by spraying the liquid on the top of their tongues. To match the intranasal protocol, subjects administered the OT/PLC as 6 individual 0.1ml puffs, one every 30s. They were instructed not to swallow during the 30s after each puff in order to give the OT an opportunity to be absorbed by lingual blood vessels. The OT and PLC spray bottles used in the study were identical to those used for intranasal administration and supplied by Sichuan Meike Pharmaceutical Co. Ltd, Sichuan, China. The PLC spray contained identical ingredients (glycerine and sodium chloride) other than OT. In post-treatment interviews subjects could not identify better than chance whether they had received OT or PLC. Thirty minutes after treatment blood samples (10 ml) were collected again to examine changes in OT blood levels following the oral treatment and then subjects underwent the implicit face-emotion processing fMRI paradigm. A validated implicit face-emotion processing task that has been previously demonstrated a sensitivity to intranasal OT was employed^8^. Briefly, the event-related paradigm incorporated 208 grayscale facial stimuli displaying happy, neutral, angry or fearful facial expressions (n = 26 per category, 50% female face stimuli). Each face was displayed on the screen for 3s and during the task subjects were required to judge the gender of the faces by button press to ensure attentive processing after the emotional faces disappeared. Following the fMRI assessment subjects rated emotional valence, arousal and intensity of each of the stimuli within 10 seconds (see Figure. S3). To control for unspecific mood changes the Positive and Negative Affect Schedule (PANAS)^9^ was administered before and after treatment as well as after finishing the task paradigm (see Table S3).

**
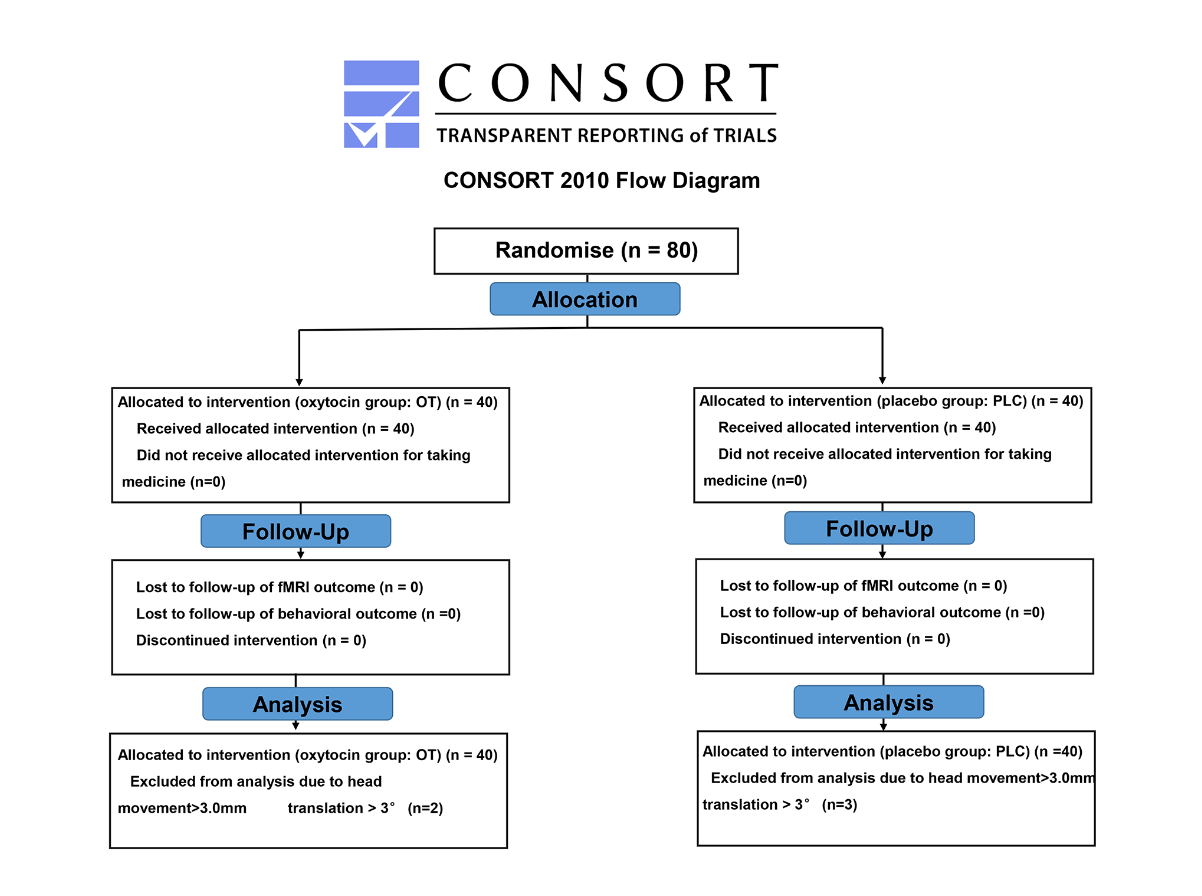
**

**Figure S1** CONSORT flow diagram


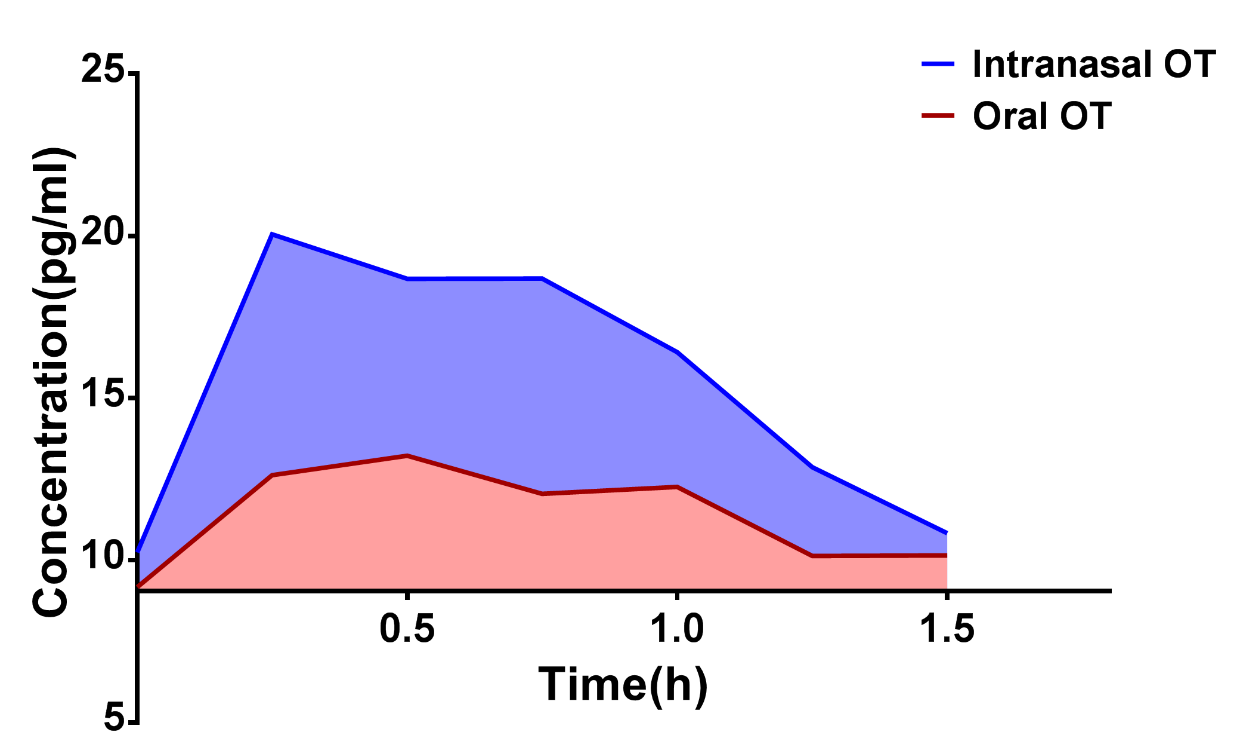


**Figure S2** Mean plasma concentration-time profiles from 0 to 1.5 hours for a single 24 IU dose OT administered via oral or intranasal routes.


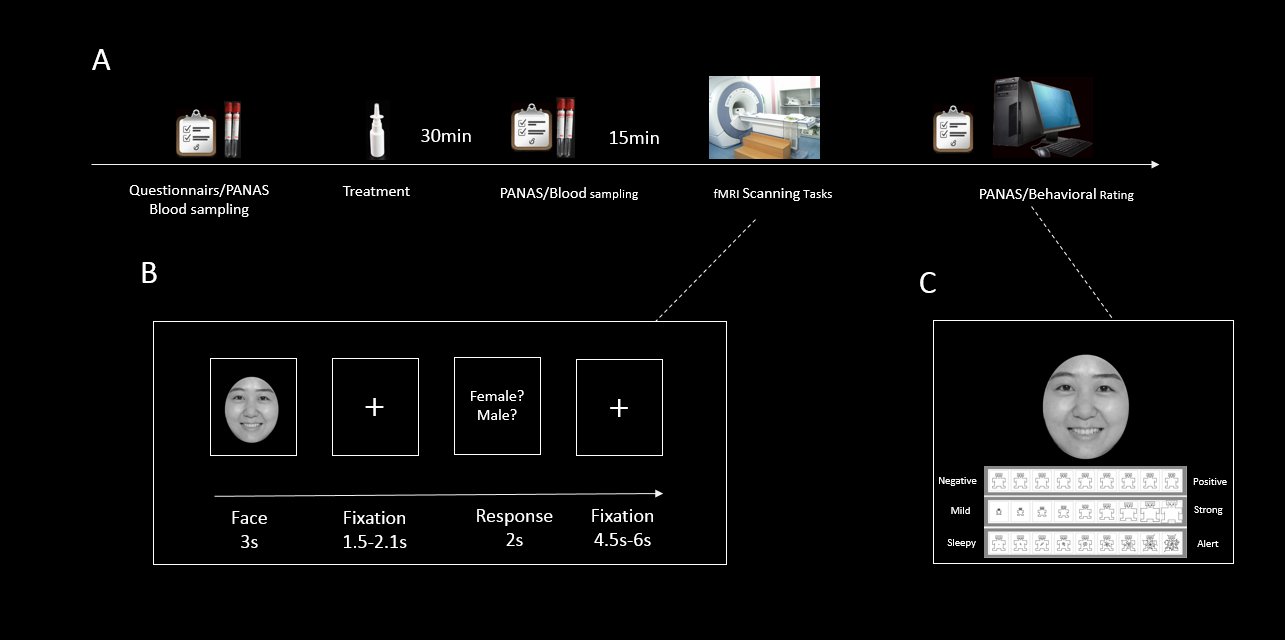


**Figure S3** Experimental protocol of oral OT part (A) experimental flow chat (B) implicit face emotional processing task in fMRI scanner (C) behavioral rating tasks out of scanner. For copyright reasons, permission to use the face picture shown as an example was obtained in writing from the individual concerned.

**Table S1** Pharmacokinetic parameters for oxytocin after intranasal or oral administration

| Route | Subject N | Cmax(pg) | Tmax(h) | | AUCt (pg.h.ml^-1^) |
| --- | --- | --- | --- | --- | --- |
| Intranasal | 15 | 20.05 | 0.25 | 8.949 | |
| Oral | 10 | 13.22 | 0.50 | 3.732 | |

**Table S2** Age, pre-treatment anxiety, depression, autism and empathy scores in two oral groups.

| Measurements | OT  n=38 | PLC  n=37 | *t* | *p* |
| --- | --- | --- | --- | --- |
| Age | 21.7±1.9 | 22.0±2.0 | 0.66 | 0.51 |
| STAI, TAI | 38.2±7.9 | 39.8±9.7 | 0.79 | 0.44 |
| STAI,SAI | 33.6±8.1 | 34.2±8.6 | 0.33 | 0.74 |
| BDI | 6.6±7.5 | 7.4±8.1 | 0.45 | 0.66 |
| AQ | 18.4±5.7 | 19.6±5.6 | 0.94 | 0.35 |
| SRS | 50.1±18.5 | 55.8±24.3 | 1.15 | 0.26 |
| IRI | 50.6±11.8 | 51.4±8.7 | 0.34 | 0.73 |
| CTQ | 36.3±8.4 | 34.8±6.4 | 0.87 | 0.39 |
| LSAS-Fear | 21.0±12.7 | 22.6±13.0 | 0.52 | 0.61 |
| LSAS-Avoidance | 19.5±10.4 | 20.6±10.7 | 0.46 | 0.65 |

**Table S3** Positive and negative mood assessment using PANAS in both groups

| Measurements | Time  point | OT  n=38 | PLC  n=37 | *t* | *p* |
| --- | --- | --- | --- | --- | --- |
| PANAS  (Positive) | Pre treatment | 24.3±7.8 | 23.8±8.2 | 0.30 | 0.8 |
|  | Post treatment | 22.6±7.4 | 22.3±8.1 | 0.16 | 0.9 |
|  | After fMRI | 20.2±7.3 | 20.4±7.7 | 0.11 | 0.9 |
| PANAS  (Negative) | Pre treatment | 11.2±4.0 | 10.9±2.9 | 0.43 | 0.6 |
|  | Post treatment | 10.1±2.3 | 9.6±1.7 | 1.03 | 0.3 |
|  | After fMRI | 9.9±2.4 | 10.0±2.4 | 0.19 | 0.9 |

References:

1 Kvaal, K., Ulstein, I., Nordhus, I. H. & Engedal, K. The Spielberger State-Trait Anxiety Inventory (STAI): the state scale in detecting mental disorders in geriatric patients. *Int. J. Geriatr. Psychiatr.* 2005; **20**: 629–634.

2 Baker, S.L., Heinrichs, N., Kim, H.J. & Hofmann, S. G. The Liebowitz social anxiety scale as a self-report instrument: a preliminary psychometric analysis. *Behav. Res. Ther.* 2002; **40**: 701–715.

3 Beck, A.T., Steer, R.A. & Carbin, M.G., Psychometric properties of the Beck Depression Inventory: Twenty-five years of evaluation. *Clin. Psychol. Rev.* 1988; **8**: 77–100.

4 Baron-Cohen, S., Hoekstra, R. A., Knickmeyer R. & Wheelwright S. The Autism-Spectrum Quotient (AQ)—Adolescent Version. *J. Autism Dev. Disord.* 2006; **36**: 343–350.

5 Bruni T. P., Test Review: Social Responsiveness Scale–Second Edition (SRS-2).  *J. Psychoeduc. Assess.* 2014; **32**: 365–369.

6 Siu, A. M. H. & Shek, D. T. L. Validation of the Interpersonal Reactivity Index in a Chinese Context. *Res. Soc. Work Pract.* 2005; **15**: 118–126.

7 Bernstein, D. P., Ahluvalia, T, Pogge, D. & Handelsman, L. Validity of the Childhood Trauma Questionnaire in an Adolescent Psychiatric Population. *J. Am. Acad. Child Adolesc. Psychiatry* 1997; **36**: 340–348.

8 Kou, J. *et al.* A randomized trial shows dose-frequency and genotype may determine the therapeutic efficacy of intranasal oxytocin. *Psychol. Med.* 2020; 1–10.

9 Watson, D., Clark, L. A. & Tellegen, A. Development and validation of brief measures of positive and negative affect: the PANAS scales. *J. Pers. Soc. Psychol.* 1988; **54**: 1063–1070.
